# Implementing asthma management guidelines in public primary care clinics in Malaysia

**DOI:** 10.1038/s41533-021-00257-5

**Published:** 2021-11-29

**Authors:** Ai Theng Cheong, Ping Yein Lee, Sazlina Shariff-Ghazali, Hani Salim, Norita Hussein, Rizawati Ramli, Hilary Pinnock, Su May Liew, Nik Sherina Hanafi, Ahmad Ihsan Abu Bakar, Azainorsuzila Mohd Ahad, Yong Kek Pang, Karuthan Chinna, Ee Ming Khoo

**Affiliations:** 1 Department of Family Medicine, Faculty of Medicine and Health Sciences, Universiti Putra Malaysia, Serdang, Selangor Malaysia; 2 UM eHealth Unit, Faculty of Medicine, University of Malaya, Kuala Lumpur, Malaysia; 3 Malaysian Research Institute on Ageing™, Universiti Putra Malaysia, Serdang, Malaysia; 4 NIHR Global Health Research Unit on Respiratory Health (RESPIRE), Usher Institute, The University of Edinburgh, Edinburgh, UK; 5 Department of Primary Care Medicine, Faculty of Medicine, University of Malaya, Kuala Lumpur, Malaysia; 6 Hospital Pusrawi Sdn Bhd, Kuala Lumpur, Malaysia; 7 Klinik Kesihatan Lukut, Ministry of Health Malaysia, Port Dickson, Negeri Sembilan Malaysia; 8 Department of Medicine, Faculty of Medicine, University of Malaya, Kuala Lumpur, Malaysia; 9 School of Medicine, Faculty of Health and Medical Sciences, Taylor’s University, Subang Jaya, Malaysia

**Keywords:** Health services, Health policy

## Abstract

Implementing asthma guideline recommendations is challenging in low- and middle-income countries. We aimed to explore healthcare provider (HCP) perspectives on the provision of recommended care. Twenty-six HCPs from six public primary care clinics in a semi-urban district of Malaysia were purposively sampled based on roles and experience. Focus group discussions were guided by a semi-structured interview guide and analysed thematically. HCPs had access to guidelines and training but highlighted multiple infrastructure-related challenges to implementing recommended care. Diagnosis and review of asthma control were hampered by limited access to spirometry and limited asthma control test (ACT) use, respectively. Treatment decisions were limited by poor availability of inhaled combination therapy (ICS/LABA) and free spacer devices. Imposed Ministry of Health programmes involving other non-communicable diseases were prioritised over asthma. Ministerial policies need practical resources and organisational support if quality improvement programmes are to facilitate better management of asthma in public primary care clinics.

## Introduction

Asthma is a common chronic respiratory disease globally, including in low- and middle-income countries (LMICs)^1^. It affects all age groups and about 235 million people are living with asthma worldwide^1^. Good asthma control is the goal of asthma management, enabling patients to carry out their normal activities^2^. Although globally, asthma control is reported to be poor^3,4^, there is variation according to regions. High-income countries such as Australia, United Kingdom and United States have reported rates of about 50% of patients with asthma as being well controlled^5–7^. In the Asia-Pacific region, only 7.6% of patients with asthma aged ≥12 years were well controlled^4^.

International evidence-based guidelines such as the Global Initiative for Asthma (GINA) reports provide recommendations for asthma management, which are based on high quality evidence^2,8^. This includes the use of objective measurements (e.g. spirometry) to confirm diagnosis, provision of spacers for those with poor inhaler technique and provision of action plans^2^. Echoed in national guidelines^9^, adherence to evidence-based recommendations would improve asthma care, reduced morbidity and mortality^10^. However, implementation may be particularly challenging in settings with limited resources such as LMICs.

Malaysia is a multi-ethnic country located in the Asia-Pacific region with a population of 32.8 million^11^. Estimates of the prevalence of asthma in the adult population range from 3.4 to 7.5%^12,13^ and prevalence in children aged 6–7 years and adolescents aged 13–14 years is 5.8 and 8.9%, respectively^14^. Only 6% of patients with asthma aged ≥12 years had well-controlled asthma^4^. The healthcare system in Malaysia includes services provided by both the public and private health sectors^15^. Public health facilities are highly subsidised by the Ministry of Health^16,17^. For example, in public primary care clinics, the fee for a clinic consultation (including cost of investigations and medications) is between RM1 to RM5 (USD 0.30–1.20), and free for government servants and pensioners, school children and people aged ≥60 years. In contrast, private primary care clinics operate on a fee for service model.

Care for chronic diseases such as diabetes, hypertension and asthma are mainly based at public primary care clinics^18^, resulting in a high workload. Routine review of chronic diseases was the commonest reason for encounters in the public primary care clinics in West Malaysia and the second in East Malaysia^18^. With limited resources, it may be challenging for these busy clinics to provide guideline-recommended standards of care. The objective of this study is to explore the challenges and potential enablers faced by healthcare providers (HCPs) in public primary care clinics in Malaysia for providing asthma care.

## Methods

### Study design

This was a qualitative study using a constructivist approach that acknowledges the inherent subjectivity and researcher’s involvement in the interpretation of the data^19,20^.

### Study settings and participants

The study was carried out in six public primary care clinics from a semi-urban district of Selangor, Malaysia during 2019. These clinics provide care of acute and chronic illness as well as maternal and child health. Asthma care is within their scope of chronic disease care.

We purposively sampled HCPs who had clinical involvement in asthma management i.e. family medicine specialists, medical officers, medical assistants, nurses, pharmacists and assistant pharmacists. Their roles are described in Table 1.

**Table 1:** Roles of HCPs in asthma management in public primary care clinics.

HCPs	Training and roles
Family medicine specialist	• Graduates of a 4-year Masters level specialist family medicine training • Lead and organise asthma care in the clinic • Serve as consultant for other HCPs to refer to if any clinical problems arise
Medical officers	• Medical graduates • Assess status of asthma control • Prescribe medication and counselling on asthma self-management
Medical assistants	• Registered medical assistants and graduates of a 3-year diploma medical assistant training • Person in charge of the treatment room, where various procedures are performed and acute emergency management initiated before referring to the doctor for further evaluation
Nurses	• Registered nurses and graduates of a 3-year diploma nursing training • Assist in assessment of asthma, such as performing vital signs and peak flow measurement • Arrange appointment for review
Pharmacists	• Qualified pharmacists • Assess and counsel patients on medication adherence • Counsel patient on inhaler technique • Counsel patient on asthma self-management
Assistant pharmacists	• Graduates of a 3-year diploma assistant pharmacist training • Dispense medication • Refer patients to pharmacists for counselling when indicated such as patients with frequent refill of prescription

### Data collection

We obtained contact information of HCPs involved in the management of patients with asthma from the Family Medicine Specialists in charge of the six public primary care clinics. Potential participants were approached via telephone call, text messages or email and invited to participate in the study. The purpose of the study was explained to the HCPs and a participant information sheet was sent to them to understand the study. For those who agreed to participate in the study, an appointment was made for a focus group discussion at which written consent was obtained. A focus group provides an opportunity for participants to interact and exchange ideas to stimulate further thoughts^21^. Each focus group had four or five participants who shared similar roles and job scope in asthma management.

Prior to the group discussion, participants completed a sociodemographic questionnaire that provided the context for the data analysis. Focus groups were held in the six clinics or meeting rooms in the district health office. A semi-structured interview guide (Supplementary Note 1), informed by literature on implementing guidelines was used to assist and facilitate discussion about participants’ experiences of management and challenges faced in delivering asthma care. Opinions were explored without restriction from the guide. Each session was moderated by a researcher (A.T.C., S.S.-G. or P.Y.L.) with the help of a note taker. The focus group discussions were conducted in English or Malay or a mix of Malay and English according to the preferences of the participants. All the moderators were bilingual. Focus groups lasted 64 min on average (range 53–77 min). All sessions were audio-taped with consent.

Data collection and analyses were performed in an iterative manner until no further new themes emerged. Recruitment was stopped after six focus group discussion when researchers agreed that the analysis had reached thematic saturation.

### Analysis

Data were analysed using thematic analysis with inductive approach^19^. All audio-recordings were transcribed verbatim in the original languages (Malay, English or a mix of Malay and English) to preserve semantics as much as possible and these served as source data for the analysis. Quotes used in papers and reports were translated into English (See Supplementary Note 2 for the original and translated quotes presented in the ‘Result’ section). Descriptors and participant numbers were used to preserve confidentiality. Data were organised using the qualitative data management software QSR NVivo 12 to facilitate analysis. Field notes with observations during the sessions and reflections at the end of discussion sessions informed the analysis.

The three researchers (A.T.C., P.Y.L., S.S.-G.) involved in the analysis are bilingual (English and Malay). The initial transcript was coded by two researchers (A.T.C. and P.Y.L.) independently. Concepts generated from the coding were then examined and compared. Similar concepts were merged into themes and subthemes and formed the initial coding frame used by A.T.C., S.S.-G. and P.Y.L. to code remaining transcripts. The coding frame was modified and reconstructed as new concepts and themes were generated and refined in discussion between the three researchers. In the event of disagreements, the original transcript and concepts were revisited and discussed to reach consensus.

All researchers were mindful that their experience as academic and primary care physicians meant that they would have preconceived ideas about delivering asthma care. Reflexivity throughout the research process during data collection and analysis helped the researchers to acknowledge and minimise their personal influence on the analysis.

In addition to allocating time to frequent discussions among the research team and revisiting analysis in the event of disagreement, a summary of the results was presented at a stakeholder engagement workshop at which HCPs and patients from participating clinics were invited to feedback. This helped to assure the quality and trustworthiness of the analysis and ensure the results correctly depicted the challenges and the situation faced in the clinics. The representatives at the workshop agreed with our conclusions.

### Ethics approval

This study received ethical approval from the Medical Research and Ethics Committee of the Ministry of Health, Malaysia (NMRR ID: NMRR-18-2683-43494) and sponsorship approval from the Academic and Clinical Central Office for Research & Development (ACCORD) at the University of Edinburgh. All participants provided written informed consent.

## Results

### Participants

Twenty-six HCPs (family medicine physicians (*n* = 5), medical officers (*n* = 5), medical assistants (*n* = 4), pharmacists (*n* = 4), assistant pharmacists (*n* = 4) and nurses (*n* = 4) were interviewed in six focus groups.

The HCPs all had access to guidelines and had received training, but there were multiple infrastructure challenges that interfered with implementing recommended care. Five themes and related subthemes are summarised in Table 2 and described below.

**Table 2:** Themes and subthemes of the results.

Theme	Subtheme
(1) Challenges in organising asthma care services with limited infrastructure	• Struggles in organising asthma care within existing clinic models and available resources • Lack of priority for asthma care compared to other established national programmes
(2) Challenges related to diagnostic assessment and review of asthma control	• Limited objective tests available for asthma diagnosis and review of control • Challenge in performing peak expiratory flow measurement • Competency of allied health staffs in assessment of acute asthma attacks
(3) Challenges in delivering asthma treatment plans	• A need for more options of drug therapy and availability of spacer devices • Challenge patient adherence to treatment • Challenges in delivering asthma education and written asthma action plans
(4) Challenges related to clinic review	• Patients only attend the clinic when symptomatic • A need to strengthen handover of care from hospital emergency department to primary care clinic • Lack of non-attenders tracing systems
(5) Challenges of training HCPs in asthma management	• The training on self-management education and counselling on written asthma action plan only included HCPs who were in charge of the asthma clinics • The training for nurses and medical assistants needed to address their roles in asthma management

### Challenges in organising asthma care services with limited infrastructure

Establishing and maintaining a health programme (such as asthma care) in a primary healthcare setting depended on the availability of human and structural resources, clinic organisation as well as support from policymakers.

It was challenging to organise and implement asthma care services at the clinic level due to multiple programmes competing for the same human resources and facilities. The clinics adopted different models for policy-driven primary care services such as the family doctor concept model and integrated clinic model.

In the family doctor concept, the HCPs were divided into teams and each team managed patients from a defined area so that people were seen by the same team at every clinic review. In the integrated clinic model, patients had the convenience of a greater choice of times for their review but not necessarily with the HCPs they knew. Both these models took up limited resources and capacity for organising a dedicated asthma clinic.

Only one of the clinics had implemented a dedicated asthma clinic for patients with partly controlled and uncontrolled asthma and found it beneficial in asthma management. This clinic ran once a week with an asthma team of designated medical officers, nurses and pharmacists. Asthma patients were registered, assessed, managed, and counselled at a ‘one-stop’ clinic review. Tracing of non-attenders (defaulters) was available for patients followed up under the dedicated asthma clinic.

“*… in terms of doctors, our* [number of] *doctors also not enough. But we try to have different programme on different days, …we feel* [dedicated asthma clinic] *is beneficial so that’s why we still carry on* [the dedicated asthma clinic] *… we got the defaulter tracing also, very good. Because they have* [registered] *the patients coming on that day, so we know who is not coming. If integrated* [clinic] *you don’t even know who did not turn up, now we know. That’s what actually happened to our clinic*.” Family medicine specialist

Though the participants agreed that having a dedicated asthma clinic could facilitate asthma care, they felt that the once-a-week dedicated clinic might not fit with the clinic organisation. To have a dedicated asthma clinic for each team in the family doctor concept model, or to run a dedicated clinic daily in the integrated clinic model would not be feasible because of constraints of manpower and consultation rooms. In addition, there were too many programmes that would need to be run by the clinics with limited resources.

“*… another restriction for my clinic is actually space. I don’t have any room to do that* [to have a dedicated asthma clinic].” Family medicine specialist

Unlike other non-communicable diseases (NCDs) such as diabetes, hypertension and mental health, there is no national asthma programme nor enforcement from policy level. National programmes have well-structured standard operating procedures with regular monitoring for quality improvement. Asthma care was thus given less weight compared with diseases under the national programme. Some clinics had initiated an asthma registry and clinical audit on their own initiative but these were not done optimally. Participants expressed that asthma was a neglected disease that deserved more attention from the Ministry of Health.

“*Just maybe can say asthma is a neglected disease*.” Family medicine specialist

It was suggested that there was a need for enforcement from higher authority and to include asthma care as a key performance index of the clinic. This would help to enhance commitment from the HCPs in delivering better asthma care.

“*Totally agree with her* [about listing asthma as a key performance index by policy makers]*. Because she said s*imilar to *diabetic clinic* [listed in key performance index]*, the MOs* [medical officer] *will try to achieve target of the performance indicator, you know*, [if] *patient did not have fundus* check, *must get it done or else the boss will audit later. All the thing, it’s just like this* [if there is a performance indicator that needs to be achieved, it will be done]. *For asthma, unfortunately there is no one to look after* [no one monitoring as no indicator that is needed to achieve]*.*” Medical officer

### Challenges related to diagnostic assessment and review of asthma control

Assessment contributes to the initial diagnosis, as well as regular review of asthma control. The challenges faced included lack of objective diagnostic tests (such as spirometry), issues in performing peak expiratory flow measurement and competency of allied health staff in carrying out assessments.

The primary care clinics did not have in-house spirometry, so the diagnosis of asthma was based on clinical history and patients’ response to prescribed medication. If there was a need to differentiate chronic obstructive pulmonary disease from asthma, patients would be referred to the hospital for spirometry assessment.

HCPs assessed patients’ asthma control status based on reported symptoms and asthma diary for those self-monitoring at home. One doctor noted discrepancies in the recording of symptom assessment between doctors and suggested templates could prompt structured assessment and documentation of symptoms.

“*…. it’s not, it’s not something you can do blood test and know this patient has achieved the target… It’s not like that. So everyone puts entry* [document] *‘good control’. Day time symptoms all recorded as none. Recorded as none. But when you ask* [the patient] *back, actually everything is there* [daytime symptoms are present].” medical officer

Examples were cited of conditions (such as diabetes) where the patient has a specific record book with templates structure for assessment, identifiable by colour; no such standard record is available for asthma.

“*…. But like diabetes we have the green book. So when* [patient] *attends* [clinic]*, the green book is retrieved. So we know, okay, we have this book, so can review, we can monitor him, from the beginning how’s the control of the sugar was. But for asthma there is no such book available.*” Medical officer

HCPs acknowledged the importance of performing peak expiratory flow assessment during acute care and in a review to establish the ‘best peak flow’. Some clinics relied on doctors to perform the assessment during consultation while others allocated the task to nurses or medical/physician assistants prior to doctor’s consultation. However, for various reasons the measurement of peak expiratory flow was often missed. These included staff forgetting to carry out the assessment, turnover of staff who were unaware of the procedure and clinic structural issues such as limited space to perform the measurements, mouthpieces not available and misplaced peak expiratory flow meter at the point of care.

“*… sometimes equipment, you know, and the PEFR need to be done there and then, the mouthpiece is not there… it is a busy day and sometimes the patient is there, but I couldn’t find the stuff [*peak flow metre] *… I say it’s okay… never mind, it’s okay.*” Medical officer

Patients with acute shortness of breath were often assessed by the medical/physician assistant in the treatment room and management initiated promptly. There was concern expressed about the competency of allied health staff in assessing patients with acute asthma attacks and there were cases reported of heart failure that had been misdiagnosed as acute asthma.

“*…, I have one incident whereby the patient told the receptionist that he is asthmatic, so the receptionist straight away instruct patient to go to the treatment room, actually uh* [it was] *cardiac asthma. … so, after the incident we told them* [allied health staff] *if they are not sure* [of diagnosis], *at least auscultate by the doctor first even if you* [allied health staff] *didn’t do the peak expiratory flow. Because some people* [allied health staff] *they didn’t ask the doctor to auscultate, the staff* [allied health staff] *in treatment room straight away give the nebulizer.*” Family medicine specialist

### Challenges in delivering asthma treatment plans

The public primary care clinics needed better support to enable them to deliver asthma treatment to achieve symptom control and health education so that the patient understands and adheres to the prescribed management.

There was a need for improved availability of medications including spacer devices to enhance the efficiency of the medication. Lack of proper spacer devices was cited as a major issue for patients with poor inhalation technique.

“…[need] *addition of budget so we can actually afford to buy better drugs, if not more quota of the existing drugs and then also probably provide a small portion of the budget to provide patients with spacer devices. It’s quite costly, it can cost about 100 ringgit, okay… previously we were actually supplied with a very cheap looking device, plastic, even FOC* [free] *by xx*[pharma] *but they have stop production of that device. So now on the worst-case scenario when the patient can’t afford to buy, we teach them to DIY* [do it yourself] *using mineral water bottle at house, so it will be really good if they can actually give a small portion of the budget to buy spacer devices*” Pharmacist

The medications that could be prescribed in the primary care clinic included inhaled corticosteroids (ICS), inhaled short acting (SABA) and long acting β_2_-agonist (LABA), oral steroids and theophylline. There was a limited supply of inhaled combination therapy (ICS/LABA), which was reserved for patients whose asthma was difficult to control. However, the demand was high and in circumstances where there was no quota available, patients who needed this medication for treatment optimisation would need to be referred to a hospital. Apart from the barrier this imposes for the individual patient needing care for their poorly controlled asthma, these referrals potentially overwhelm the hospital clinics when it can still be managed in primary care and increase overall costs.

“*… but I feel that more people should be allowed to use better medication to manage their asthma because some of them come there, come back very frequently for oral steroids, yea so if given these type of inhalers, better inhalers [combination ICS/LABA]* *I think their asthma management would be much better, so we don’t have to depend so much on oral steroid…*” Pharmacist

For acute exacerbations of asthma, nebuliser therapy was provided in the health clinic. There was concern regarding the old nebuliser machines that were inefficient in delivering the therapy.

“*I* [have] *four* [nebulisers]*. Out of these four, one and two often have problems. Sometimes it’s not working. One of them the suction is very slow. Ahh… so the medicine was not released. Patient also complaint they did not feel [*the treatment effect]*.*” Staff nurse

HCPs understood the importance of gaining patients’ confidence and adherence to the treatment plan to achieve treatment goals, though our participants reported difficulty in ensuring patient adherence because of patients’ negative perceptions towards corticosteroid side effects especially the risk of addiction.

“*…., but again, compliance ah is always the case, when they are getting a bit better or there’s some false believe that the ICS can get addiction. Some of my patients say they do not use it regularly. They are afraid of being addicted and are unable to do without the ICS also. These are some factors, patient’s taboo and believe, false belief yeah.*” Family medicine specialist

Asthma education was recognised as an important component of asthma management, and despite challenges, HCPs would provide information about asthma and teach patients how to manage their asthma. However, there was no allocated time for asthma education in contrast to other diseases such as diabetes.

“*So, for me, I feel the management for asthma is not like for diabetes. Diabetes has a special class. Its* [asthma] *education is lacking. We want to educate patients, but do not have time, and then there is no specific day for us to deliver the education.*” Medical officer

There was concern over the lack of good health education materials that are user-friendly and easy to understand by patients to facilitate the counselling process. There is a need to have the materials in multiple languages (Malay, English, Mandarin and Tamil) to accommodate the multi-ethnic patients who attend the clinics.

“*… we do not really have good education material whereby we are trying to empower the patient so that they can really know what the preventer is for and what is the deliver for. even though we are supposed to counsel them, pharmacist supposed to counsel them.*” Family medicine specialist

Written asthma action plans were provided by the family medicine specialists, medical officers or pharmacists, though this was new to some of the medical officers and pharmacists and not all clinics implemented action plans. For those who had counselled patients on action plans, there were practical issues of limited available copies of written plans and concerns about patients’ abilities to understand and follow the recommendations.

“*So those patients were actually able to grasp what you are saying, no other problems so I’m sure to give it* [written action plan] *to them. Not on the first session itself maybe the second or third session depending on the performance….No, not all patients* [been given an action plan] *because I don’t want it to back fire if they do the wrong thing and it’s going to get them into trouble, that’s worst.*” Pharmacist

Challenges were identified in delivering asthma education to children and older patients. Study participants noted that the education was directed towards the carers rather than the patients and this could contribute to the poor compliance and asthma control.

“*Um problem, there are problems with children and elderly. They [elderly] do not know how to inhale, sometimes the one who listens to the counseling is his child. For children, the mother or father is the one who listens, not the child. Ahh, what this mean is, the counseling is not delievered to the person who uses the inhaler, the patient…*” Assistant pharmacist

### Challenges related to clinic review

Regular asthma review by HCPs is important for optimisation of management, continuity of care and is especially important after an acute attack.

It was often found that patients only attend the clinic when symptomatic. HCPs reported that patients did not attend the recommended early review after an out-of-hours or hospital emergency attendance or for regular scheduled appointments.

“*We treat them accordingly and then, we, usually we ask them to come back in a week or two, to re-assess the symptoms, whether need to step up the treatment* [after exacerbation]. *Usually, so far, what I’ve seen they rarely come back. They only come back when their inhaler is empty or when, and they come back when they have another attack.*” Medical officer

There is a need to strengthen the handover of care from the hospital emergency department to primary care clinic for continuity of care after an acute exacerbation. Though there is a protocol, our participants in primary care doubted that this was implemented as planned. And considered that typically patients would be treated for the acute attack without being given a scheduled clinic review.

“*That’s some more, patients going to ED* [emergency department]*, won’t be referred properly to us, sometimes they just get the nebulizer and just go off.*” Family medicine specialist

Tracing of non-attenders is a measure to improve scheduled review. In primary care practice, this system was not well established except in the one clinic that implemented a dedicated asthma clinic.

HCPs acknowledged the importance of non-attenders tracing but limited manpower running multiple concurrent programmes were among the reasons that hindered its implementation.

“*… for dedicated clinic we have some opportunity to trace the defaulter. cause uh, we actually quite fortunate because our team is very dedicated as well and they have, they are spending some times to call the defaulters but that is for the dedicated clinic, okay*,” Family medicine specialist

“*I think it’s a really ideal idea for defaulter tracing but, we are lacking manpower. so that is the main problem. If possible, we want to do that part. However, our staff is basically multitasking. Yeah, everything with the same staff, same manpower, so that’s why we cannot cope*.” Family medicine specialist

### Challenges of training HCPs in asthma management

The HCPs had access to guidelines and had attended training for asthma management in the clinic and at district level. Some HCPs had attended training at state or national level. Although the training was perceived as sufficient for the doctors and pharmacists, the training for nurses and medical assistants also needed to address their roles in asthma management. In addition, the training on self-management education and counselling on written asthma action plan only included HCPs who were in charge of the asthma clinics.

“*…and after coming back from the district training, the KK* [clinic] *level, every session of the CME* [training course] *we will ask those who have attended the district course to brief the other MO [medical officer] who have not had the opportunity to attend. That’s why I believe the training part is not too lacking…*” Family medicine specialist

“*Ahh no, to be honest I’m not aware of this* [asthma action plan] *cause I’m not actually in charge of the respi MTAC* [respiratory medication therapy adherence clinic]*.*” Pharmacist

## Discussion

There were a range of practical challenges faced by HCPs in public primary care clinics in implementing guideline-recommended care at all stages of asthma care, from organising the programme, assessment, treatment and routine review. Having multiple NCD programmes to manage with limited resources and lack of infrastructure were highlighted as underpinning many of the challenges to meeting asthma management standards and contributes to short- and long-term inequities in healthcare provision in asthma diagnosis, treatment and follow-up.

We have included all category of HCPs who were directly involved in asthma care in public primary care clinics in the study. This had provided a better understanding regarding the challenges faced by the HCPs at primary care level. The data collected had reached saturation and analysis was done in original language to retain nuances. Patients’ views were not included in this article. However, their input was included to support interpretation of the results. The six centres included were from public sector and urban area and the findings might not be representative of centres elsewhere (e.g. clinic from rural area) in Malaysia and challenges faced by the private general practitioners.

Our participants highlighted challenges that were multifactorial and included healthcare patient, professional and organisational barriers. This resonates with findings from previous literature, which highlighted the importance of targeting all these factors with the initiatives promoted and supported by policies in order to improve the provision of asthma care^22,23^.

Although highlighted as a priority NCD by the World Health Organisation^1^, respiratory disease is consistently underfunded and overlooked compared to the priority to reduce cardiovascular risk and diabetes within health systems^24^. This was reflected in the prioritisation of diabetes and cardiovascular care at the clinic level and the challenges of implementing structured asthma care in accordance to guideline recommendation^25^. Despite the limited resources, however, one of the clinics in our study had managed to organise a dedicated asthma clinic for patients with uncontrolled asthma. This was achieved through efficient organisation of manpower and multidisciplinary effort i.e. involvement of the pharmacist for patient education. Despite the benefits of a dedicated asthma clinic that could focus on delivering systematic assessment and management of asthma^26^, insufficient resources precluded re-structuring of asthma care in the other participating clinics, which prioritised other national programmes.

In common with many LMICs^27,28^, spirometry was not available in the primary care setting in our study, even though measurement of lung function is important in primary care settings to avoid under- or over-diagnosis of asthma. It has been suggested that clinical prediction models may support the diagnosis of asthma in primary care, but to date, existing models are unreliable to inform practice^29^, and patients needing lung function testing (e.g. to rule out chronic obstructive pulmonary disease) have to attend hospital. Without proper assessment, there is a risk of under treating patients with asthma or exposing patients without asthma to unnecessary treatment at primary care^30^.

Peak expiratory flow measurement is important and reliable for assessment of asthma severity and response to treatment^2,9^. In Malaysia, home monitoring of peak expiratory flow measurement is not a common practice. Thus, office measurement of peak expiratory flow during follow-up might help to ascertain patients’ personal best peak expiratory flow reading and facilitate monitoring of asthma control. If home monitoring of peak expiratory flow rate could be adopted by patients, it could help in early recognition and prompt treatment of asthma exacerbations^31,32^. Low usage of peak expiratory flow meter had been reported by both the HCPs and patients in other LMICs^27^. Peak expiratory flow meters were available in our studied clinics but not at the point of assessment.

Regular review of asthma control by healthcare professionals is important to improve asthma outcomes^2,33^. There were care gaps in the primary care management of asthma in meeting the guideline recommendations such as structured assessment of asthma control at each visit^34^ and providing appointments for regular reviews^35–37^. Although not unique to LMICs (a practice audit from Canada reported that symptom-based questions were asked in 6.2% of asthma consultations and only 15.4% of patients had their control status determined^34^), lack of formal documentation as used in diabetes care challenged routine assessment and recording of asthma control. Questionnaire such as asthma control test (ACT) or questionnaire from GINA are recommended for estimating asthma symptoms control^2,9^. Routine administration of ACT during clinic reviews could reduce the gap between the patient’s experience of specific symptoms and the physician’s general report of control especially in patients with a lower level of education^38^.

Our participants were concerned that patients were rarely reviewed in primary care following an acute asthma exacerbation at hospital emergency department. A systematic review has shown that educational interventions targeting either patients or emergency department HCPs could improve follow-up with primary HCPs after exacerbations^37^. In addition, patient reminders are important to increase the adherence to regular routine reviews^39^.

Asthma education programmes that enable patients to understand their asthma, promote adherence to medication and correct inhalation technique, self-monitor their condition, supported by regular review of asthma control by healthcare professionals and guided self-management using asthma action plan could improve asthma outcome^33,40^. Our participants had observed misconceptions among patients about inhalers, which affected adherence and poor inhalation techniques. In addition, spacers to facilitate effective use of pressurised metered-dose inhalers (pMDI) were unavailable in public primary care clinics. Funding for other devices (such as dry powder inhalers) or spacers at public primary care clinics is crucial to overcome issues around pMDI inhalation techniques. A local study found that a high proportion (71.9%) of adult patients in a public primary care clinic had a poor inhaler technique^41^, which might have been improved with the use of a spacer especially for the older population^42^. In the absence of commercially produced spacers, the pharmacists in our study practices taught patients using do-it-yourself spacers as there is some limited evidence that this may be an effective strategy^43,44^.

A recent systematic review has reported that regular supported self-management (which involves an investment of at least 2 h of support from HCPs) was effective in reducing healthcare use and improved quality of life especially in patients with mild-to-moderate symptoms of asthma^45^. The time constraints of the HCPs we interviewed mean that this will be difficult to achieve.

Our findings echo concerns that, although chronic respiratory diseases are one of the WHO prioritised NCDs^46^, compared to cardiovascular disease, diabetes and cancer they are the least likely to have national policies^47^, community-based care and referral strategies or be the focus of research^24^. Asthma (or other chronic respiratory diseases) are not even mentioned in the most recent Malaysian National Health and Morbidity Survey^48^. Overcoming the organisational barriers that we identified will require national recognition of the importance of chronic respiratory disease, appropriate policies to enable cost-effective delivery of guideline recommended care and regular surveillance of their impact.

Good communication among HCPs and teamwork contributed to effective delegation of works and organised asthma care within the practices, which could improve implementation of guideline recommendations^25^. Each clinic could develop an asthma care pathway with tasks designated to appropriate HCPs. In addition, there is a need for better communication and handover of care from hospital emergency departments to primary care clinics to enable continuity of asthma care and reviews of asthma severity and treatment after critical events.

A paper-based or electronic template with a structured questionnaire would prompt a structured assessment of asthma control^49^. Patients could be provided with an asthma control questionnaire to complete before seeing the doctor, fitting in with existing routines as it involves only minimal change to the current care pathway.

With the high usage of internet and mobile applications such as smartphone among the Malaysian population^50,51^, other potential options in the near future will be the use of interactive digital applications with a symptom checklist that patients could complete before seeing their physicians, reducing assessment time in a busy primary care clinic. In addition, reminders of appointment could be incorporated, which could help improve attendance at regular review.

The investment of time required to support self-management might be feasible with involvement of other HCPs such as pharmacists, nurses or medical assistants, in addition to the doctors^52^. HCP training on education required and completing asthma action plans would need to be strengthened to improve delivery of supported self-management to the patients.

There is a need to look at the shared care model between public and private primary care providers for more even distribution of the workload^15,53^ and the opportunity to expand resources. The support and initiative at policy levels will be key for implementing a new reimbursement scheme for the private primary care doctors to share the workload for chronic disease care^54^.

Challenges faced by the HCPs in delivering guideline recommended asthma care in the primary care setting requires attention to patients’ needs, professionals’ training and organisational initiatives to structure the asthma care pathway. Limited resources and lack of infrastructure were highlighted as specific challenges for meeting asthma management standards. There is a need for policies at national/ministry level to make respiratory disease care a national priority to address these issues.

### Reporting summary

Further information on research design is available in the Nature Research Reporting Summary linked to this article.

## Supplementary information

Reporting Summary

Supplementary Information

## Data Availability

The data are not publicly available due to them containing information that could compromise research participant privacy.
