# Supplementary material for: Implementing asthma management guidelines in public primary care clinics in Malaysia
Source: NPJ Prim Care Respir Med. 2021 Nov 29;31:47. doi: 10.1038/s41533-021-00257-5 (PMC8630037; doi:10.1038/s41533-021-00257-5)
Supplement: Supplementary file 2 — Supplementary Information [file 41533_2021_257_MOESM2_ESM.pdf]

Supplementary Note 1

Semi-structured interview guide for HCPs

Preamble:

- Ice-breaking and explain the aim of the study
- Explain that there is no right or wrong answer
- Explain need to get consent for the interview and audio-recording

Question	Prompts
1. How do you manage asthma in your clinic?	• What do you assess for the control?<br>• Any guideline? Any local protocol?<br>• Asthma program?
2. How do you decide on the treatment plan for the patients?	• Pharmacological<br>• Non-pharmacological
3. What do you think about the service provided by your clinic to the asthma patients? Any problems?	• Facilities<br>• human resource (asthma team),<br>• medications

Supplementary Note 2. Original quotes presented in the result section

Original quotes	*Translated quotes*
"Just maybe boleh cakap neglected la kan asthma ni neglected disease. "	*"Just maybe can say asthma is a neglected disease."*
"Totally agree with her [about putting asthma as a key performance index by policy makers]. *Because she said macam like diabetic clinic, macam the MOs* [medical officer] *will kejar, you know, patient tak buat fundus, kena buat fundus nanti boss akan audit. All the thing, macam memang ni. Kalau asthma memang there is no one to look after."*	*"Totally agree with her* [about listing asthma as a key performance index by policy makers]. *Because she said* similar to *diabetic clinic* [listed in key performance index] *, the MOs* [medical officer] *will try to achieve target of the performance indicator, you know,* [if] *patient did not have fundus* check, *must get it done or else the boss will audit later. All the thing, it's just like this* [if there is a performance indicator that needs to be achieved, it will be done]. *For asthma, unfortunately there is no one to look after* [no one monitoring as no indicator that is needed to achieve]*."*
" .... it's not, it's not something you boleh ambil darah, patient ni dah achieve target... It's not like that. So everyone put entry good control. Day time symptom semua takde. Put there nil. Padahal when you ask back, actually everything is there."	*".... it's not, it's not something you can do blood test and know this patient has achieved the target... It's not like that. So everyone puts entry* [document] *'good control'. Day time symptoms all recorded as none. Recorded as none. But when you ask* [the patient] *back, actually everything is there* [daytime symptoms are present]*."*
"... But like diabetic we have the green book. So [pesakit] datang je green book. So tahu, okay, we have this book, so boleh tengok, kita boleh monitor dia, from the beginning how's the control of the sugar. But for asthma takde."	*".... But like diabetes we have the green book. So when* [patient] *attends* [clinic], *the green book is retrieved. So we know, okay, we have this book, so can review, we can monitor him, from the beginning how's the control of the sugar was. But for asthma there is no such book available."*
"Saya [ada] empat [nebulizer], daripada empat tu, satu dan dua selalu buat masalah. Kadang-kadang dia rosak. Satu tu dia slow sangat suction dia tu kan. Ahh.. so ubat dia tak- tak keluar. Patient pun complain tak rasa."	"*I* [have] *four* [nebulisers]. *Out of these four, one and two often have problems. Sometimes it's not working. One of them the suction is very slow. Ahh ... so the medicine was not released. Patient also complaint they did not feel [*the treatment effect]."
".... , but again to compliance ah is always the case, when uh they are getting a bit better or there's some false believe that the ICS can get addiction la. Some of my patienst say they	*"...., but again, compliance ah is always the case, when they are getting a bit better or there's some false believe that the ICS can get addiction. Some of my patients say they do*

do not use it regularly dia takut nak addicted, ah, dan dia tak boleh tinggalkan the ICS also. These are some factors, patient's taboo and believe, false believe yeah."	*not use it regularly. They are afraid of being addicted and are unable to do without the ICS also. These are some factors, patient's taboo and believe, false belief yeah."*
"So, For me I rasa macam management untuk asthma ni macam, bukan macam kencing manis lah. Kencing manis ada class khas. Dia[Asthma] punya education lacking sikit. Kita nak educate patient pun kita takde masa, and then takda specific day untuk kita bagi education."	*"So, for me, I feel the management for asthma is not like for diabetes. Diabetes has a special class. Its* [asthma] *education is lacking. We want to educate patients, but do not have time, and then there is no specific day for us to deliver the education."*
"Um masalah, kalau tengok kanak kanak dengan orang tua yang kadang-kadang masalah. Dia [orang tua] *tak tau nak sedut, kadang kadang dengar kaunseling tu anak dia, kalau budak, mak atau bapa dia yang dengar,tapi anak dia tak dengar. Ahh tu maknanya apa ni, penyampaian tu tak kepada orang yang mengguna ubat inhaler tu, pesakit tu…"*	*"Um problem, there are problems with children and elderly. They* [elderly] *do not know how to inhale, sometimes the one who listens to the counseling is his child. For children, the mother or father is the one who listens, not the child. Ahh, what this mean is, the counseling is not delievered to the person who uses the inhaler, the patient… "*
